# Novel Therapeutic Agents for Management of Diabetes Mellitus: A Hope for Drug Designing against Diabetes Mellitus

**DOI:** 10.3390/life14010099

**Published:** 2024-01-08

**Authors:** Ahmed M. E. Elkhalifa, Mehak Nazar, Sofi Imtiyaz Ali, Ibraq Khursheed, Syed Taifa, Muzafar Ahmad Mir, Iqra Hussain Shah, Masood Malik, Zahid Ramzan, Shubeena Ahad, Nusrat Bashir, Elham Elamin, Elsharif A. Bazie, Elsadig Mohamed Ahmed, Majed Mowanes Alruwaili, Ammar W. Baltoyour, Abdullah Salah Alarfaj, Ibrahim Ali Al Bataj, Abdullah M. A. Arabe, Showkat Ul Nabi

**Affiliations:** 1 Department of Public Health, College of Health Sciences, Saudi Electronic University, Riyadh 11673, Saudi Arabia; a.alkhalifa@seu.edu.sa; 2 Department of Haematology, Faculty of Medical Laboratory Sciences, University of El Imam El Mahdi, Kosti 1158, Sudan; lhmelamin@yahoo.com; 3 Preclinical Research Laboratory, Department of Clinical Veterinary Medicine, Ethics & Jurisprudence, Sher-e-Kashmir University of Agricultural Sciences and Technology (SKUAST-Kashmir), Srinagar 190006, India; mehaknazar2422@gmail.com (M.N.); imtiyazali@skuastkashmir.ac.in (S.I.A.); syedtaifa786@gmail.com (S.T.); mahimuzaffar111@gmail.com (M.A.M.); iqrahussainshah@gmail.com (I.H.S.); drmasoodmalik16@gmail.com (M.M.); zahidramzan367@gmail.com (Z.R.); shubeenaahad786@gmail.com (S.A.); nusratbashir510@gmail.com (N.B.); 4 Department of Zoology, Central University of Kashmir, Nunar, Ganderbal 191201, India; seemawani111@gmail.com; 5 Pediatric Department, Faculty of Medicine, University of El Imam El Mahdi, Kosti 1158, Sudan; elsharifbazie@gmail.com; 6 Department of Clinical Chemistry, Faculty of Medical Laboratory Sciences, University of El Imam El Mahdi, Kosti 1158, Sudan; 7 Department of Medical Laboratory Sciences, College of Applied Medical Sciences, University of Bisha, P.O. Box 551, Bisha 61922, Saudi Arabia; 8 Nursing Administration & Education Department, College of Nursing, Jouf University, Sakaka 72388, Saudi Arabia; majed@ju.edu.sa; 9 Dhahran Eye Specialist Hospital, Ministry of Health, Dhahran 39455, Saudi Arabia; abaltoyour@moh.gov.sa; 10 Dr. Suliman Al-Habib Hospital, Riyadh 11372, Saudi Arabia; 11 Najran General Hospital, Ministry of Health, Najran 66277, Saudi Arabia; b3taaj@hotmail.com; 12 Diabetic Center and Unites, Ministry of Health, Riyadh 11176, Saudi Arabia

**Keywords:** diabetes mellitus, therapeutics, novel drugs, stem cell, immunotherapeutic and phytotherapy

## Abstract

Diabetes mellitus (DM) is characterized by an absolute decline in insulin secretion and peripheral resistance and is the most prevalent metabolic and endocrine disorder. However, the pathogenesis of DM also includes adipocyte insulin resistance, increased glucagon secretion, increased renal glomerular glucose absorption, and neurotransmitter dysfunction. Although there is a wide spectrum of therapeutics available for glycemic control, owing to the identification of various pathogenic determinants of DM, management of DM remains challenging and complex. Current therapeutic interventions against DM focus mostly on glycemic control without considering the other pathological determinants that eventually lead to treatment failure and the progression of DM. Furthermore, long-term use of these conventionally available anti-diabetic drugs leads to various side effects, henceforth development of novel drugs against DM remains an unending search strategy for researchers. Various studies conducted in various parts of the world have proposed that these novel therapeutic interventions target multiple and alternate pathogenic hotspots involved in DM. The current review article discusses novel therapeutic options that hold particular promise to support their safety and discuss the side effects resulting from their use so that these novel candidate drugs can be effectively fabricated into potential drugs for the treatment of DM.

## 1. Introduction

India being the “Diabetes metropolis” of the planet influences almost every community whether it is metropolitan or agrarian [1]. Diabetes mellitus (DM) is characterized by hyperglycemia resulting from insulin resistance, inadequate insulin secretion, or excessive glucagon secretion. DM is a non-infectious disease caused by the disruption of carbohydrate metabolism, which culminates in persistent hyperglycemia [2,3,4]. According to the World Health Organization (WHO), DM will be the seventh prime cause of death in 2030 [5]. The International Diabetes Federation (IDF) has figured that by 2021, 536.6 million will be living with diabetes, and by 2045, this figure is forecasted to elevate by 46%, reaching 783.2 million [6]. However, the microvascular (stroke, cardiovascular ailment, and peripheral artery disease) and macrovascular (nephropathy, neuropathy, and retinopathy) problems are the two main chronic complications of the disease with the former having more presence than the latter [6]. Owing to the high prevalence of the disease, the poor health consequences and surge in the costs of treatment make it obligatory to focus on early diagnosis with the consequent alterations in the management of DM [6].

With reference to insulin deficiency, DM has been grouped into type 1 diabetes mellitus (T1DM) also known as insulin-dependent diabetes mellitus/juvenile-onset diabetes associated with the autoimmune destruction of pancreatic beta cells and is present in 5–10% of diabetic patients [1]. These patients require insulin shots for the maintenance of blood glucose levels and are associated with other autoimmune disorders like autoimmune hepatitis, grave’s disease, vitiligo, Addison’s disease, myasthenia gravis, pernicious anemia, and Hashimoto’s thyroiditis [7]. T1DM is hereditary in nature and the occurrence rate of T1DM differs globally, depending on the interplay between genetic susceptibility and various environmental factors. Lately, it has been reported that increased mortality and morbidity rates for TIDM were allied with low socio-demographic status [8,9] Type 2 diabetes mellitus (T2DM) also recognized as insulin-nondependent diabetes mellitus (NIDDM)/mature-onset diabetes is the fourth main reason for deaths in various developed countries along possibilities for cardiac ailments like coronary heart disease and stroke [10]. Idiopathic diabetes covers genetic susceptibility and the requirement for insulin replacement therapy in affected patients is needed of an hour. Some patients of Oriental or Nigerian lineage with T1IDM possess hardly any causative factor, however, remain susceptible to the occurrence of diabetic ketoacidosis with insulinopenia [1]. Catamenial hyperglycemia is unimpeded hyperglycemia that is coincident with diabetic ketoacidosis and usually occurs before the commencement of the menstrual cycle in women, hence called catamenial diabetic ketoacidosis [11]. The persistent hyperglycemia results in an increased insulin requirement usually up to four times [12]. However, the pathophysiology still remains unknown [13]. Gestational diabetes mellitus (GDM) is observed during pregnancy and occurs due to glucose intolerance subsequently resulting in hyperglycemia with severity levels of variable degrees [14]. It is diagnosed during parturiency, influencing 14% of parturient women, and within the US influences 135,000 women yearly [15,16]. A new entrant called “Latent Autoimmune Diabetes in Adults” (LADA) has been added to the diabetes mellitus spectrum [17]. LADA shares characteristics of both T1DM and T2DM, henceforth it has been referred to as T1.5DM. The characteristic features of the disease include an autoimmune response where the body’s immune system attacks and destroys the insulin-producing beta cells in the pancreas, adult onset, gradual onset, and insulin dependency [18]. Recently, researchers have attributed LADA as a sequel of coronavirus disease (COVID-19), where viral determinants of severe acute respiratory syndrome coronavirus 2 (SARS-CoV-2) play a role in triggering the autoimmune reaction [19]. It is important for individuals with LADA to work closely with healthcare professionals to manage their condition effectively [20,21].

Conventional treatment of DM involves lifestyle modifications, oral medications, and insulin therapy. Lifestyle modifications include regular physical exercise, dietary management, and weight loss, and lifestyle modification has been reported to be more effective in the management of T2DM compared to T1DM [22]. There is a plethora of oral anti-diabetic drugs available that target various pathogenic hotspots involved in hyperglycemia [23]. The most commonly used oral hyperglycemic drugs include biguanides, which specifically reduce hepatic glycogenolysis and improve peripheral insulin sensitivity [24]. Sulfonylureas are drugs that act on pancreatic β cells and cause the release of insulin [25]. Similarly, thiazolidinediones improve insulin sensitivity in peripheral tissues [26]. For patients who cannot achieve glycemic targets through lifestyle modification and oral hypoglycemic drugs, insulin therapy has been advocated [27].

Recently various novel approaches have been adopted for the management of DM, which include nanotechnology-based insulin delivery systems based on improving the precision, efficiency, and patient compliance in insulin therapy [28]. Nanoparticles for glucose monitoring, for instance, gold nanoparticles and quantum dots have unique optical and electrical properties that can be harnessed for the development of sensitive glucose sensors [29]. Smart insulin delivery system innovations are based on advanced technologies to provide precise and automated control over insulin administration [30].

## 2. Current Anti-Diabetes Therapeutic Regimens

The available pharmacotherapy for the treatment of DM includes insulin and various oral hypoglycemic agents like sulfonylureas, metformin, α-glucosidase inhibitors (acarbose and troglitazone), and pioglitazone/rosiglitazone [31] (Table 1 and Table 2).

Additionally, thiazolidinedione insulin sensitizers, peroxisome proliferator-activated receptors, glucagon receptor antagonists, and dipeptidyl peptidase IV inhibitors are being used as therapeutic regimens to manage DM [36]. However, these current standard drugs do not restore normal glucose homeostasis for long periods and are associated with adverse consequences like hypoglycemia, kidney diseases, GIT problems, hepatotoxicity, heart risk problems, and insulinoma, which necessities the need for the development of an alternative therapeutic regimen for the management of DM [37]. A comprehensive table of the most commonly used conventional therapeutic agents for the amelioration and cure of diabetes briefing their merits and demerits is given in (Table 1, Table 3 and Table 4).

**Figure 1:**
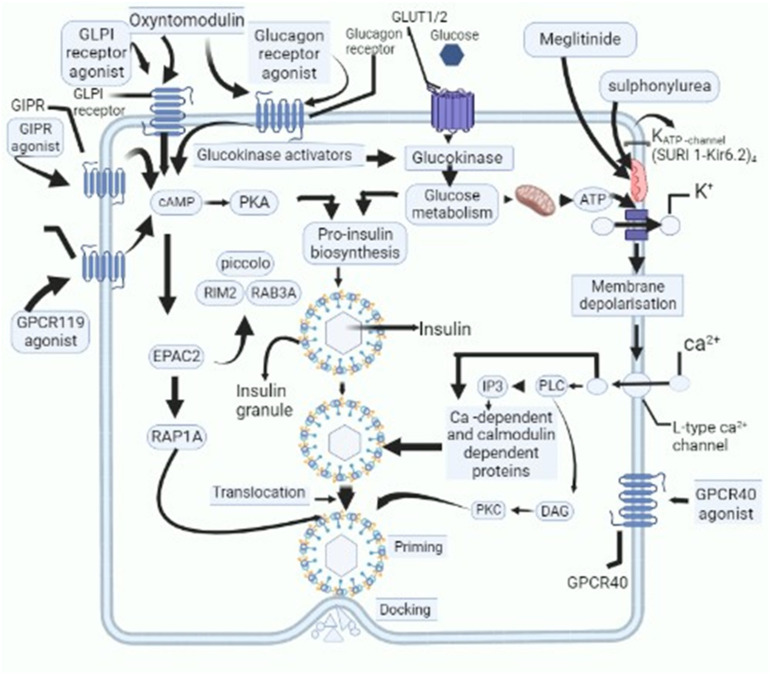
Schematic outline of cellular activities of novel candidate drugs proposed to be used as anti-diabetic drugs and their action on various cellular and subcellular structures, which cause decline in levels of blood glucose. GPR119-G protein-coupled receptor-119; gastric inhibitory polypeptide receptor: GIPR-; glucagon-like peptide-1: GLP-1-; glucose transporters: GLUT; cyclic adenosine monophosphate: cAMP; protein kinase A: PKA; Ras-related protein Rap-1A: RAP1-A; exchange protein activated by cyclic-AMP: EPAC2; Ras-related protein Rab-3A: RAB-3A; inositol trisphosphate: IP3; diacylglycerol: DAG; protein kinase C: PKC; phospholipase C: PLC.

Nowadays, considerable attention is being laid down on the development of novel therapeutic drugs for the management of DM [40]. For instance, medicinal plants and their bioactive compounds have been found to have a variety of therapeutic actions against DM, which include effects on insulin synthesis, increased insulin secretion, amelioration of peripheral resistance, dampening of chronic inflammation associated with DM, and scavenging free radicals [41]. Researchers across various parts of the world are working on the development of personalized medicinal preparations and have proposed various novel preparations against DM [42], the list is ever-increasing and in some cases, the mechanism of action of these formulations has been identified (Figure 1).

However, the majority of these studies are at an early stage, and pathogenic hotspots targeted by these novel formulations are very ill-understood. In the current study, an extensive and comprehensive review has been drafted keeping in view the development of novel drugs proposed by various studies. The manuscript is sought to provide comprehensive information on these novel formulations. The findings of this review will give impetus to identifying the most novel regimens for the management of DM and elucidating their mechanisms of action at the cellular level and subcellular levels (Table 5).

## 3. Novel Therapeutic Agents

Current understanding of the pathophysiology of DM from the triumvirate of β cell failure to “ominous octet” has identified multiple pathogenic hotspots in the pathogenesis of DM [12]. Likewise, recognition of the “ominous octet” in the pathogenesis of DM has provided insight into the development of novel therapeutic agents against DM [51]. In a subsequent section of this manuscript, we have discussed the novel therapeutic agents against DM that can be used in the future for effective management of DM, some of these therapeutic agents are in phase 3 of clinical trials and some are in the preclinical phase of development (Table 5 and Table 6).

### 3.1. Stem Cell Therapy: An Emerging Arrow for Targeting Diabetes Mellitus

**Figure 2:**
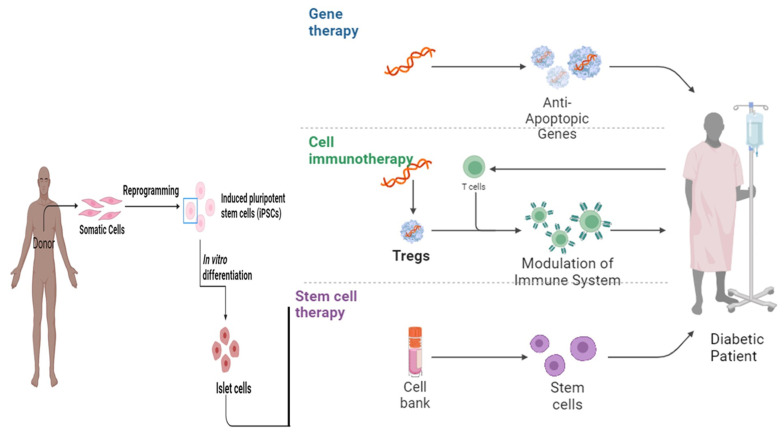
The therapeutic landscape: exploring the role of gene therapy, cell immunotherapy, and stem cell therapy in diabetes mellitus [58].

Various scientists working in stem cells established therapeutics pertaining to T1DM via the production of full-grown β cells originating from stem cells [48]. However, it becomes necessary to acquire the type and number of stem cells required for the treatment of the disease as it is an established fact that the pancreas does not have the capability of regeneration [51]. Embryonic stem cells (ESc) have the potential to differentiate into cells mimicking insulin secretagogue activity and various in vitro and in vivo studies have confirmed that the transformation of ESc into insulin-like cells results in the improvement of glucose uptake and metabolism [55,56]. Similarly, intravenous (IV) injection of embryonic-like stem cells (VSELs) in mice with pancreatic necrosis showed the potential to repair the damaged pancreas in diabetic patients [57] (Figure 2).

A smart biological system developed from mesenchymal stem cells isolated from the Wharton jelly component of the umbilical cord covered with immunoisolatory microcapsules was observed to restore the β cell population of T1DM patients [59]. Furthermore, significant elevation of the C-peptide stratum was observed after implantation of mesenchymal stem cells in six T2DM patients isolated from the umbilical cord. Consequently, postprandial stability in the serum glucose levels was noticed after 2 h [60]. To advance further, adiponectin, an adipocyte-secreted adipokine was observed to control the mobilization of bone marrow-derived mesenchymal stem cells (BMSCs). Adiponectin assisted in BMSC migration from the bone marrow into the circulation to regenerate bone by regulating stromal cell-derived factor (SDF)-1 in a mouse bone defect model and importantly lowered glucose levels and encouraged bone regeneration in mice with diet-induced obesity [32]. Similarly, stem cells labeled with positron emission tomography (PET) tracer fluorine 18-fluorodeoxyglucose (F-FDG) to evaluate satisfactory administration methods for these cells in diabetic patients resulted in homing and retention of stem cells in the pancreas. Furthermore, infusion into the superior gastroduodenal artery (superior branch) was found to be the preferred route into the splenic artery as the former method resulted in better homing and retention of labeled stem cells [31]. The mesenchymal stromal cells with the insulin-secreting property isolated from the adipose tissue conjointly with the hematopoietic stem cells extracted from bone marrow, co-infused into the thymic portal circulation and subcutaneous tissue were found to regulate the hyperglycemia in TIDM patients [61]. Subsequent to the incorporation of human eyelid insulin-secreting stem cells (derivatives of adipose tissue), there was a lowering of serum glucose level in T2DM by increasing the insulin level in circulation [62].

The activity of the autoimmune mechanism underlying T1DM can be suppressed by immune ablation. Likewise, for immune ablation, 24 patients underwent transplantation of autologous hematopoietic stem cell transplantation (AHSCT) with a high dose of cyclophosphamide and anti-thymocyte globulin. From these studies, it was found that AHSCT leads to a remission of T1DM with good glycemic control [63]. Furthermore, the possible risk factors identified for rejection of AHSCT therapy include levels of C-peptide (fasting), age, and the levels of TNF-α [49]. In the later half of the 19th century, the attention of scientists was directed towards somatic cell-derived pluripotent stem cells (induced). The efficacy of somatic pluripotent cell lines/induced cell lines was reported as a healing technique for T1DM [52,64]. For instance, following the implantation of the altered epithelial cells isolated from the pancreas of non-obese diabetic mice within diabetic mice transformed precisely into insulin-producing cells with further significance in beta cell markers of the pancreas in addition to boosting the insulin release induced by glucose and potassium chloride [64]. To be more precise and accurate, Pancreatic stem cells were evaluated as therapeutic tools for amelioration of DM. In this direction, the intravenous injection of pancreatic stem cells in T1DM patients isolated from fetuses resulted in remarkable elevation of C-peptide levels after 3 months of intravenous injection of pancreatic stem cells [60].

### 3.2. Transdermal Drug Delivery System (TDDS)

The primary treatment regimen for the management of diabetes still remains the oral hypoglycemic drugs along with insulin injections [65]. Nevertheless, in the last 10 years, the TDDS has received considerable attention as an alternative regimen for the amelioration of diabetes due to its beneficial effects in comparison to the oral forms and injections, which are usually invasive along with being painful [66]. Other than carrying drugs such as insulin and metformin, the TDDS works by analyzing the metabolism via biosensing by evaluating metabolites in biological fluids like sweat [43]. In this direction, ref. [63] designed a biosensor patch by incorporating a microneedle array (3D) for monitoring blood glucose levels. Furthermore, in vitro experiments indicated its stability in long-term use and the potential to check glucose levels even at extreme values. One of the limitations found in the experiment was that the sensitivity in detecting glucose levels decreased as glucose levels increased due to bio-fouling around the electrodes used. Hence, further improvement in the design is required to address this issue.

The development of effective transdermal systems faces challenges but also holds promise for improved patient compliance and therapeutic outcomes [67]. The technological approaches adopted under TDDS include microneedle technology. Under this technology, tiny needles enhance drug delivery by creating microchannels hence improving the permeability of antidiabetic drugs. Likewise, nanoformulations have been used for TDDS of various anti-diabetic drugs, insulin sensitizers, and insulin [68]. Furthermore, innovative techniques, which include iontophoresis and electroporation, utilize electric fields for the penetration of anti-diabetic drugs and insulin [61]. The advantages these approaches offer include non-invasiveness, steady and prolonged release, avoidance of first-pass metabolism, and reduced systemic side effects [30]. As research progresses, the translation of these technologies into clinically viable and widely accepted options remains an exciting avenue for improving diabetes care.

### 3.3. Nanotechnology

It is well understood that insulin injections used conventionally for TIDM and T2DM are accompanied by painful dispensing and infections which are associated with subsequent low patient care [69]. Hence, to overcome these obstacles, the nanotarget perspective is undertaken, which is gaining tremendous popularity in the present era for being accurate, specific, efficacious, and favorable [14]. Henceforth, nanotechnology has been widely used for the management of DM due to the miniaturization of glucose sensors and closed-loop insulin delivery systems [70]. Accordingly, smart nanoparticles (NPs) as drug delivery systems contain glucose sensors, which help in sensing the glucose level in the body and accordingly help with insulin transportation. These bioengineered molecules contain microcapsules with pores that are small enough to permit the transit of insulin [44]. These nanoparticle formulations have been found to have greater drug bioavailability along with the fact that maximum drugs could be delivered at specific targets. However, their expandability and noxious nature can prove to be dangerous [16]. For instance, a nanotechnology-based insulin delivery system offers precise targeting of pathogenic hotspots involved in the pathogenesis of DM at minimal doses, which improves the pharmacokinetics of insulin with reduced side effects [38]. Furthermore, quantum dots and mesoporous silica nanoparticles, have been employed to develop highly sensitive and selective glucose sensors [47]. Likewise, the integration of nanotechnology in smart insulin delivery systems allows for glucose-responsive insulin release [30]. Despite the significant strides in nanotechnology for diabetes management, challenges such as biocompatibility, long-term safety, and scalability must be addressed for clinical translation.

### 3.4. Novel Candidate Drugs for Management of DM

#### 3.4.1. Fucoidan

The aquatic ecosystem is well known for the origin of nutraceuticals, cosmetics, and agronomic compounds (24). The various biologically active metabolites isolated from seaweeds (green algae, red algae, and brown algae) have been described to possess a wide range of pharmacological properties [4,7,71,72]. For instance, fucoidan obtained from sea cucumbers is a revolutionary biological sulfated polysaccharide [70,72]. Similarly, *Chorda filum*, *Fucus evanescens*, *Hizikia fusiforme*, *Sargassum stenophyllum*, *Laminaria hyperborean*, *Caulerpa racemosa*, *Analipus japonica*, *Fucus serratus*, *Padina gymnospora*, *Ascophyllum nodusum*, *Fucus vesiculosus*, and *Kjellmaniella crassifolia* have been explored for their fucoidan composition and have gained enormous interest for being agents of diabetes amelioration along with the amelioration of other metabolic diseases. Similarly, fucoidan isolated from the Fucus vesiculosus acts as a glucosidase inhibitor and thus plays a role as an anti-diabetic agent [5,12,72]. Additionally, fucoidan has the capability of reducing diabetic retinopathy by the inhibition of VEGF (vascular endothelial growth factor) signaling [41]. In addition to this, in preclinical studies, fucoidan has been used for the management of diabetes via the alleviation of symptoms associated with the disease [15,37,53,73]. Fucoidan likely alleviates hyperglycemia by regulating activated protein kinase (AMPK) signaling together with GLUT-4 action (34). Notably, Fuc-Pg, a fucoidan derived from the *Pearsonothuria graeei* (molecular weight -310 kDa) can be employed as a functional agent for the treatment of many metabolic disorders [74,75]. Moreover, Fuc-Pg was found to be responsible for the reduction of weight apart from decreasing hyperlipidemia and protecting the liver from steatosis in high-fat-diet-fed mice [66,76].

#### 3.4.2. SGLT-2 (Sodium–Glucose Transporter-2) Inhibitors

Sodium–glucose transporter (SGLT-2) inhibitors, a distinct Na-glucose transporter expressed by epithelia presented around renal proximal tubules comprise one futuristic therapeutic category for T2DM management. They are in greater numbers (around 90%) in the kidney tubular epithelium unlike SGLT-1 isoforms mostly found in intestines [77,78]. They act by barring the renal tubular glucose re-absorption along with showing an insulin discrete approach. Conceptually, these inhibitors could be utilized with the inclusion of other anti-diabetic drugs like insulin [36,79]. At Present, canagliflozin, dapagliflozin, and empagliflozin are commercially used in diabetic patients. Although this class of drugs has shown benefits, chronic outcomes of using this category of drugs still need to be evaluated (Table 1) [80].

#### 3.4.3. Statin Therapy

Statins are considered novel therapeutic tools for the management and control of diabetes. Statins are categorized under 3-hydroxy-3-methyl-glutaryl-coenzyme A, commonly known as HMG-CoA reductase inhibitor [72]. Statins are recognized for the filtering of LDL (low-density lipoprotein) and consequently diminishing their level in blood accompanied by the strengthening of blood vessels [81]. Henceforth, they offer the dual advantages of preventing cardiovascular disease (CVD), the most noticeable and prominent consequence of T2DM, and the amelioration of diabetic ketoacidosis. They are familiar lipid-lowering vehicles as they act on the cholesterol genesis pathway by transforming HMG-CoA into mevalonic acids. Importantly, a clinical trial was conducted on 6000 patients given statin therapy, and the study concluded that statin therapy acts on the lipolytic pathway and hence imparts a therapeutic effect by maintaining microvascular integrity, which prevents angiopathy in diabetic patients. However, chronic use was associated with myositis, hepatic disorders, and kidney problems [10,82].

Numerous clinical trials and observational studies have consistently demonstrated the anti-diabetic potential of statins. These drugs effectively lower low-density lipoprotein cholesterol (LDL-C) levels and reduce the risk of hyperglycemia-mediated cellular and subcellular damage. However, concerns have been raised regarding the association between statin therapy and the development of new-onset diabetes mellitus. Several large-scale studies, including the JUPITER trial (Justification for the Use of Statins in Prevention: An Intervention Trial Evaluating Rosuvastatin) and meta-analyses, have reported a modest increase in the risk of developing diabetes among statin users. Henceforth, decisions to initiate or continue statin therapy should involve a careful assessment of individual age, gender, family history, and baseline glucose metabolism. Based on these assumptions, finding an individualized approach to statin therapy is crucial, taking into consideration the patient’s diabetogenic risk factors. Henceforth, regular monitoring of blood glucose levels and glycated hemoglobin (HbA1c) is recommended, especially in patients with pre-existing risk factors for diabetes.

#### 3.4.4. Quercetin Shielding against Diabetes

Quercetin [31] belongs to flavonoids and has been extracted from many fruits and vegetables like berries, onions, seeds, various nuts, barks, tea, flowers, leaves, and brassica vegetables [7,83]. Recently, pharmacological studies have shown that quercetin has biological properties relating to human health which encompasses protection against CVD, anti-allergic, anti-cancer, anti-ulcer, anti-inflammatory, anti-diabetic, and eye protection via avoidance of cataract formation [25,84]. Similarly, the role of quercetin as an antioxidant agent by inhibiting the enzyme xanthine oxidase is not clear. The greater hindrance in the utilization of quercetin is because of its lesser oral bioavailability, which is believed to be because of the presence of the sugar moiety of the molecule [72]. Quercetin has been progressively seen to decrease the complications of diabetes by acting on various signal pathways [4]. Moreover, administration of the different doses of quercetin orally in streptozotocin (STZ) and alloxan-induced diabetes rat models was capable of bringing down the blood glucose levels and glycosylated Hb (HbA1C) [9,70]. Following the oral administration of quercetin in a diabetic rat model, a significant decline in serum glucose levels was observed and the therapeutic mechanism was attributed to restoring the islet of Langerhans, boosting the insulin level in the serum along with stimulating the release of insulin. Furthermore, in T2DM mice models (C57BL/KsJ-db/db) [49] and the high-fat diet-induced insulin resistance model [85], quercetin was observed to reduce the skeletal glucose uptake with subsequently influencing the insulin secretion (glucose-stimulated). It has been hypothesized that the hypoglycemic mechanism of quercetin might be attributed to GLUT (glucose transporter) expression or enhancing the insulin signal transduction via the upregulation of gene/protein expression (Figure 2), together with the phosphorylation of the insulin receptor or insulin receptor substrate. Similarly, quercetin showed a remedial approach against the significant complication of diabetes namely diabetic nephropathy through hypoglycemic, anti-inflammatory, and anti-oxidant characteristics [80,86].

### 3.5. Immunological Approach

Nowadays, immunological therapy has gained immense attraction for treating DM, especially T1DM. Usually, there are two immunological approaches namely non-antigen-specific and antigen-specific ones [78]. The most commonly used immunomodulatory agents gaining popularity include cyclosporine A, cytotoxic T cells, anti-CD3 cells, anti-thymocyte globulin, insulin, heat shock proteins, anti-TNF, glutamic acid decarboxylase, and mycophenolate mofetil. In addition to these, studies are being conducted on dendritic cells, IL-4, IL-2, regulatory T cells, M2 macrophages, and the amalgam of IL-2 and rapamycin for evaluating their significance in amelioration of T1DM [80]. Among these agents, some of them have been evaluated for the management of T1DM in various animal models [69]. Previously, in the 1980s and 1990s, numerous clinical trials were conducted to look into the efficacy of commonly used immune-suppressive drugs (usually non-antigen-specific) in T1DM [55] with a focus on disrupting the progression and development of DM. On that note, a brief list of commonly used non-antigen-specific immunomodulatory drugs/agents has been described and explained below.

#### 3.5.1. Cyclosporin A (CsA)

Cyclosporine A (calcineurin inhibitor) has been recognized as one of the earliest and primary immunosuppressive drugs attributed to its influential immune suppressant activity against T1DM [49]. It acts via interference with the signal transduction mediated by the TCR (T cell receptors), followed by an interruption in activation of T cells along with the consequent reduction in IL-2 secretion by helper T cells [87]. Nonetheless, there are certain disadvantages of persistent usage in patients with T1DM like cost and significant toxicity of pancreatic β cells [43].

Regulatory T cells (Tregs): Regulatory T cells (Tregs) have shown promise as a potential therapeutic tool in the context of type 1 diabetes (T1D) [19,88]. Tregs play a crucial role in maintaining immune homeostasis by suppressing excessive immune responses and preventing autoimmune reactions [88]. Tregs may help to modulate the immune response to preserve beta cell function. For instance, authors have postulated the use of Tregs as a therapeutic vaccination against T1DM. Similarly, CD4(+) CD25(high)CD127- Tregs have positive outcomes in terms of pancreatic islet survival [23,89].

#### 3.5.2. Rituximab

Antigen-specific immunomodulatory drugs (monoclonal antibody) against B-lymphocyte antigen-CD-20 (surface marker), which is expressed by immature as well as mature B lymphocytes [11]. Recently, a phase II trial was conducted to observe the patency of β cells by the use of rituximab in T1DM. A total of 87 patients with T1DM were administered 4 doses/week of the said drug and one year later, it was observed that the average C-peptide AUC (area under the curve) appears to be elevated in comparison to the placebo group [36].

#### 3.5.3. Anti-TNF-α

These agents are most commonly used as therapeutics for chronic inflammatory autoimmune disorders like rheumatoid arthritis [33]. However, a double-blinded experiment using etanercept (anti-TNF-α) showed lowering the dose of insulin required in the case of children aids in the proliferation of pancreatic β cells [34]. However, recently it was also observed that the anti-TNF-α binds with the TNF-α receptor and henceforth inhibits the advancement and development of DM [33]. These agents have been found to have the potential of deactivating T cells and henceforth inhibit the apoptosis of pancreatic β cells [36].

#### 3.5.4. GAD-65 (Glutamic Acid Decarboxylase 65)

A single intranasal administration of GAD65 peptides to NOD mice induced a Th2 cell response that inhibits the spontaneous development of autoreactive Th1 responses and the progression of β cell autoimmunity in NOD mice and henceforth reduces pancreatic apoptosis and TIDM incidence [35].

#### 3.5.5. Insulin Secretagogues (TAK-875)

G protein-coupled receptor-40 (GpcR-40) is a surface receptor and it has the highest expression in pancreatic β cells. Activation of GpcR-40 by fatty acids or synthetic ligands stimulates insulin secretion, but only in the presence of elevated glucose concentrations [39]. TAK-875 is a recent addition to novel drugs, these molecules act on GpcR-40 causing hypoglycemia and significantly increasing the insulinogenic index in diabetic patients. Although the candidate molecule was showing encouraging results, owing to hepatoxicity associated with chronic use of the candidate drug, the drug was stopped after the 10th week of phase 2 randomized, double-blind, placebo- and active comparator-controlled 12-week trial [16]. However other GpcR-40 agonists are under consideration in various preclinical and clinical stages for the development of novel drugs against DM. For instance, GPR-119 agonists, which act directly on the β cell and enteroendocrine K- and L cells to increase insulin and incretin secretion, respectively, have shown promise [85].

### 3.6. Ethno-Medicine

**Figure 3:**
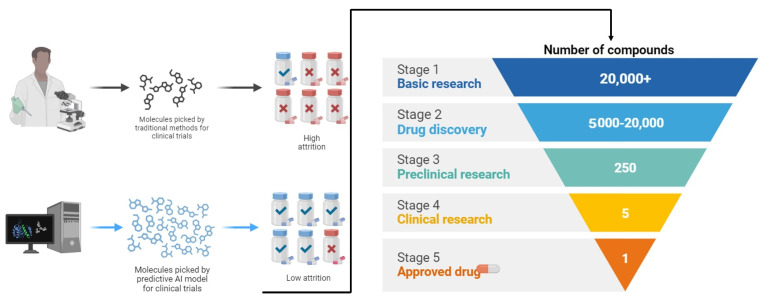
Exploration into the intricate world of drug discovery from plants and the potent bioactive compounds unearthed through botanical exploration and scientific innovation.

India is known for its traditional medicinal systems (Ayurveda, Siddha, and Unani) and is considered the garden of the world for growing a larger variety of herbs. Globally, there are around 21,000 herbs mentioned by WHO that have a broad range of pharmacological properties along with functional chemical components involved in the treatment and control of DM [27] These bioactive compounds isolated from the plant source are economically feasible, having less adverse effects along with being affordable by any class of society [80]. These bioactive compounds are employed on a larger scale due to their anti-diabetic properties viz. hypoglycemic effects in contrast to conventional drugs like metformin, tolbutamide, and chlorpropamide [49,90]. There are numerous herbal plants namely *Allium sativum*, *Gymnemasylbestre*, *Allium cepa*, *Spreng*, *Withania somniferous*, *Murraya koenigii*, etc., which have been reported to possess anti-diabetic constituents like terpenoids, flavonoids, phenolics, coumarins, and many other glucose reducing components being biologically active [31]. The mode of action of maximum herbal-based drugs is not clear despite their usage for decades all over the world [53]. A comprehensive table of different phytochemicals along with their mode of action is described in (Table 7) and their fabrication into potential drugs (Figure 3).

### 3.7. Dietetics

Diet and nutrition have always proved important and significant aspects for the management and control of DM. Medical nutrition therapy by the American Diabetes Association (ADA) is an essential component of diabetes management that comprises counseling and recommendations for dietary intake and nutrition goals by registered dieticians or nutrition experts to optimize metabolic control and maximize treatment outcomes. It includes designing diet plans individualized per patient needs along with regular monitoring by the registered dietician and diabetologist [45]. It has been observed that a low carbohydrate with high protein diet is a very effective dietary regime that is associated initially with a loss of weight followed by sustained blood sugar control. However, it is not so easy to maintain it for a prolonged period. A well-balanced and appropriate nutrition regime that works well is one with the inclusion of a high-fiber diet (HFD @ 20–35 g/day of both soluble and insoluble fiber followed by the intake of protein kept @ 10–20%, total fat needs to be constrained around less than 30% and 2400–3000 mg/day of sodium decrease with the inclusion of multivitamins [46].

**Figure 4:**
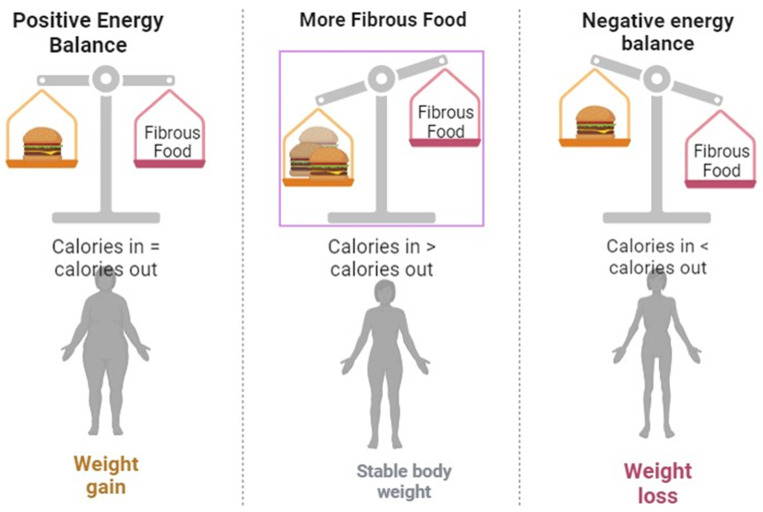
A Comprehensive guide to developing and implementing an optimal dietary plan for managing diabetes mellitus.

Nowadays, vitamin D is being used on a large scale as a supplemental drug for various metabolic syndromes along with DM. However, the importance and significance of consuming an ideal and preferable dose of vitamin D is a matter of discussion worldwide [50]. Consequently, various studies have revealed a remarkable link between a deficiency of vitamin D and the emergence of T1DM [40,54,92,102]. On examination, low exposure to vitamin D during pregnancy [93] and restricting the consumption of vitamin D-fortified food [31] has resulted in a greater probability of developing T1DM. Furthermore, the supplementation of vitamin D during the early years of childhood resulted in a decline in the advancement of DM [94]. The outcome of the supplementation of vitamin D on the emergence of T1DM appears to be dependent on the different stages of life, as the supplementation of vitamin D between 7–8 months of age has resulted in two-fold lesser chances of development of T1DM [95]. Contrarily, numerous studies have reported that there is no relationship between vitamin D supplementation and reduced incidence of DM [96,97,98,100]. Additionally, the deficiency of vitamin D appears to have an effect on insulin resistance and subsequently T2DM [32,101,103]. Figure 4 describes the general nutritional strategies and dietary recommendations for optimal health management in individuals affected by diabetes mellitus.

## 4. Conclusions and Future Perspectives

Nowadays, diabetes mellitus is gaining vast and immense attention owing to its catastrophic consequences on the human population globally. There has been a persistent search going on all over the world from all ages to combat the disease due to its huge devastating effects on the lives of human beings. For this reason, the disease needs to be continuously monitored and managed for leading and maintaining a healthy and sound life. The drugs (insulin, metformin, tolbutamide, glipizide, etc.), which were being used conventionally for a long span for the management of DM are now being substituted by better drugs owing to their potency, enhanced pharmacological action, long-lasting effects, lesser or reduced after-effects along with remarkable results on maintaining the blood glucose levels within the desired range. Moreover, the significance and importance of exercise, dynamic lifestyle, and diet for the management of the disease should not be overlooked especially in the case of T2DM. Presently, various pharmacological agents tested for the amelioration of the disease showed more promising results but have been associated with various side effects. For that reason, the disease is being evaluated on a large scale globally and phytomedicines are gaining immense attention owing to their promising effects on DM. Also, the rationale for testing phytomedicines on a large scale is due to its low cost, lesser side effects, long-lasting beneficial effects, and better patient observance in contrast to the synthetic drugs/allopathic drugs used for the management of DM. Thus, the management and control of DM is an ongoing process with a focus on finding a better therapeutic approach, keeping in mind the economics and disastrous potentiality of the mentioned disease. Correspondingly, the measures for the management of DM are shaped in contrary ways, which can be promising for controlling the epidemic of the disease on a global scale.

## Figures and Tables

**Table 1:** Commercial drugs used for management of diabetes mellitus and their associated mechanism of action with advantages and side effects.

Alpha Glucosidase Inhibitors	These Are Budding Therapeutics Aimed at Blocking α-Glucosidase, Henceforth, Postponing Breakdown of Carbohydrates Which Consequently Diminishes Its Intestinal Assimilation [5]				
Drugs	Commercial Name	Mechanism of Action	Advantages	Side Effects	References
(a) Acarbose	Precose	There is amendable blockage of glucosidases esp. glucoamylase, sucrose, maltose, and α-amylase of brush border epithelium	Decreased postprandial hyperglycemia in T2DM	Borborygmic, abdominal fullness, diarrhea, intestinal flatulence	[32]
(b) Migtitol	Glycet	Works by reducing the disintegration and assimilation of sugar in small intestine	Considerable hypoglycemia by achievement of normoglycemia in patients via enhancement of glucose tolerance	Lesser GIT after-effects	[5,27,33]
(c) Voglibose		Newly added significant sucrose blocker	Slows down the glucose assimilation, accordingly decreasing the possibility of macrovascular complications		[5]
(d) Insulin sensitizers		Aid the activity of insulin in liver, adipose tissue, and muscles in addition to decreasing the peripheral insulin resistance in skeletal muscles and adipocytes		Hypoglycemia, weight gain, diarrhea, and greater chance of cardiovascular diseases	[34]

**Table 2:** Sulfonylureas class of drugs used for management of diabetes mellitus and their associated mechanism of action with advantages and side effects.

Sulfonylureas	Sulfonylureas Are a Class of Oral Antidiabetic Drugs Commonly Used in the Treatment of Type 2 Diabetes. They Work by Stimulating Insulin Release from the Beta Cells of the Pancreas				
Drugs	Commercial Name	Mechanism of Action	Advantages	Side Effects	References
First-generation sulfonylureas					
(a) Chlorpropamide (b) Tolazamide (c) Tolbutamide (d) Acetohexamide	(a) Diabinese (b) Tolinase (c) Orinase (d) Dyemelor	These cause stimulation of the secretion of insulin from pancreas	Considerable hypoglycemia is achieved	GIT interferences and hemolytic anemia	[22]
2. Second-generation sulfonylureas					
(a) Glyburide (b) Glimepiride (c) Glipizide	(a) Glynase (b) Gleam (c) Glucotrol	All of these promote the secretion of insulin from β cells of pancreas	Considerable hypoglycemia is achieved	GIT interferences and hemolytic anemia	[5,21,35]

**Table 3:** Biguanides class of drugs used for management of diabetes mellitus and their associated mechanism of action with advantages and side effects.

Biguanides	They Primarily Work by Reducing Hepatic Glucose Production and Improving Peripheral Insulin Sensitivity			
Drugs	Mechanism of Action	Advantages	Side Effects	References
Metformin (extracted from plant Galega officinalis)	Works by improving the serum glucose levels via hindering the liver glucose production along with boosting the uptake of glucose by muscle fibers	Declining triglycerides and low-density lipids and has lesser frequency of hypoglycemia	Lactic acidosis, vit B12 deficiency, congestive heart failure	[9]

**Table 4:** Thiazolidinediones (TZD) class of drugs used for management of diabetes mellitus and their associated mechanism of action with advantages and side effects.

Thiazolidinediones (TZD)	These Drugs Work by Targeting the Peroxisome Proliferator-Activated Receptor Gamma (PPAR-γ), a Nuclear Receptor Involved in Glucose and Lipid Metabolism				
Drugs	Commercial Name	Mechanism of Action	Advantages	Side Effects	References
Troglitazone Rosiglitazone Pioglitazone	(a) Rezulin (b) Avandia (c) Actos	Enhances insulin sensitivity of all target tissues and acts as ligands for PPAR (peroxisomes proliferator-activated gamma) complex located inside the nucleus	Effect on plasma low-density lipids/cholesterol	(a) Class III–IV heart failure. (b) Fluid retention, peripheral edema (c) CHF (congestive heart failure)	[5,38,39]

**Table 5:** Novel peptide analogs class of drugs used for management of diabetes mellitus and their associated mechanism of action with advantages and side effects.

Peptide Analogues	These Analogues Are Synthetic Compounds Designed to Mimic the Actions of Endogenous Peptides Involved in Glucose Homeostasis			
Drugs	Mechanism of Action	Advantages	Side Effects	References
Incretin mimetics	Liberated in riposte to the ingestion of food, eliciting the glucose-induced insulin response	Appetence decreasing positive impact of these agents on cardiovascular, inflammation, and the central nervous system	Does not hinder the glucagon emission	[43,44]
Glucagon-like peptide analogs and agonists (GLP-1)	Retarding the gastric clearing, boosting the insulin secretion alongside hampering the glucagon secretion from pancreas	Prevents gastric acid secretion in addition to promoting insulin secretion	Not reported	[35,45,46]
Glucose-dependent insulinotropic polypeptide analogs (GIP)	These analogs are synthetic compounds designed to mimic the actions of endogenous peptides involved in glucose homeostasis	Reducing the postprandial glucose levels and glycosylated hemoglobin	Lesser danger of hypoglycemia with the use of this agent	[20,47]
DPP-4 inhibitors	GIP produced out of the k cells of the upper small intestine works by affecting the metabolism of lipids	Fasting plasma glucose levels reduced along with the changes in glycosylated hemoglobin	Effects like vomiting and anorexia	[8,12]
Liraglutide	Action via enzymatic incretin disintegration Agonist for receptor (GLP-1)	Appetence decreasing positive impact of these agents on cardiovascular, inflammation, and the central nervous system	Compared to the short-acting forms, long-acting ones have less chance of causing hypoglycemia	[9,48,49,50]

**Table 6:** Novel anti-diabetic agents used for management of diabetes mellitus and their associated mechanism of action with advantages and side effects.

Novel Anti-Diabetic Agents	The Latest Developments in Pharmacotherapy, Focusing on Innovative Approaches That Address the Complex Challenges of Diabetes			
Drugs	Mechanism of Action	Advantages	Side Effects	References
Amylin analogs (islet amyloid polypeptide	Significant decline in the levels of glucose in T1DM and T2DM	Decreases the blood glucose level by reducing the glucagon secretion	Used as solo therapy or sometimes in combination with orally active anti-diabetic agents.	[23,52,53,54]
2. Pramlintide	Secreted and stored in combination with insulin and hindering the glucagon secretion in addition to slowing down the unloading of gastric contents	If there is some beta cell function remaining, then at that time, replacement curative aided with basal insulin can prove to be useful	Increased arterial pressure, inducing kidney dysfunction, onset of hypertension, boosting the occurrence of diabetes and hypothyroidism	[23,52,53,54]
3. Nitrate/Nitrite	Molecules produced from *L-arginine* via the enzymes nitric oxide synthase (NOS) namely inducible, neuronal, endothelial, and mitochondrial have been observed to abate the levels of triglycerides in serum	Regulation of TG levels in blood	Promotion of fat deposition in liver	[23,52,53,54]
4. Antioxidant therapy: vitamin E, vitamin C, and β carotene	Fasting plasma insulin and HbA1c levels are declined by administration of vitamin C. Moreover, there is seen to be refinement of insulin action. Likewise, with the administration of β carotene, reduction of oxidative low-density lipids has been noted	New effective therapy for the cure of T2DM patients is antioxidant therapy, which might reflect a significant role in diminishing the chances of diabetic hyperglycemia and thereafter its associated drawbacks		[23,52,53,54]
5. FBPase (fructose 1,6-bisphosphatase) inhibitors	Mode of action via inhibiting the FBPase enzyme (rate-limiting enzyme in gluconeogenesis pathway)		Liver hyperplasia, liver hypertrophy, and liver carcinogenesis	[23,52,53,54]
6. Bromocriptine	Recently, Swift-liberated bromocriptine evolved in favor of T2DM amelioration but the mode of action is not clear yet	The literature has proved that after 24 weeks of therapy, the average glycated hemoglobin levels declined by 0.0% to 0.2%		[23,52,53,54]
7. Imeglimin (yet to pass the clinical trial)	Quite effective in the sense that it stimulates glucose uptake in muscles, liver gluconeogenesis is depressed along with boost in the sugar-dependent insulin release	Hampering oxidative phosphorylation		[23,52,53,54]
8. Peroxisome proliferator-activated receptors (PPARs)		Mostly participating in controlling the energy homeostasis supplementary to reduction of triglycerides		[23,52,53,54]

**Table 7:** Phytomedicinal preparations used for management of diabetes mellitus, plant parts supposed to possess high levels of pharmacologically active principles the active principles identified for their therapeutic action, and possible mechanism of action.

Phytomedicine with Family	Portion	Active Chemical Constituents	Mechanism of Action	References
*Allium cepa* (Onion) Alliaceae	Corm	S-methyl cysteine sulphoxide and allyl propyl disulfide	Arouse the action of enzymes hexokinase and reductase in addition to the production of insulin	[66]
*Carica papaya* Caricaceae	Seed and extract of leaves	------	Alleviate wounds in alloxan-induced diabetic rats in addition to reducing the serum glucose level	[37]
*Catharantus roseus* (Vinca roses) Apocynaceae	Leaves and twigs	-------	Boosting the biosynthesis of insulin from the pancreatic islets	[91]
Acacia Arabica Fabaceae	Bark and seed	Polyphenols and tannins	Commencement of insulin secretion from pancreatic β cells	[92]
*Allium sativum* (Garlic) Alliaceae	Corm	Allicin and allyl propyl disulfide	Modifies the action of enzymes glucose-6-phosphate, HMG CoA reductase, and hexokinase, in addition to managing glucose levels in serum and tissues	[51]
*Aloe barbadensis* (Aloevera/Ghikanwar) Liliaceae	Leaf	Barbaloin and alloin	Revitalizing the process of hepatogluconeogenesis/glycogenolysis alongside the liberation of insulin from the pancreas. In addition, the glutathione levels in diabetic rats were elevated by a factor of 4	[4,63]
*Beta vulgaris* (Beet root)	Root	Betacyanins and phenolics	Non-enzymatic glycosylation of serum glucose and skin proteins declined	[92]
*Azadirachta indica* (Neem) Meliaceae	Seed and leaf	Nimbin and azadirachtin	β cells of the pancreas are revived/revitalized Also, it has been noted to amend blood circulation via dilating blood vessels (Mishra et al., 2011)	[93,94]
*Brassica nigra* (Mustard) Brassicaceae	Whole plant	Sinignin, isorhamnetin, diglucoside, and isothiocynate	The conduct of glycogen synthetase is boosted unlike the action of glycogen phosphorylase and gluconeogenic enzymes, which is reduced thereby depressing glycogenolysis and gluconeogenesis	[51]
*Cassia auriculata* (Senna) Leguminaceae	Flower	Sennoside A and Sennosede B	The activity of hepatic hexokinase and phosphofructokinase enzymes is amplified while the activity of glucose-6-phosphate and fructose-1,6-biphosphatase enzymes is suppressed. Further, there is an increase in the no. of islets and beta cells in pancreas	[51]
*Andrographis Paniculata* (Kalmegh) Acanthaceae	Whole plant	Andrographolide, kalmeghin, and diterpenoid lactone	Glucose assimilation from the intestinal wall is countered	[51,62]
*Gymnema sylvestre* (Gudmar) Asclepiadaceae	Leaf	Gymnema saponins and gymnemic acid	Boosting the number of β cells along with insulin secretion	[63]
*Ficus benglenesis* (Banyan) Moraceae	Bark and leaf	Tannin, taraxasterol, quercetin-3-galactoside, and rutin	Blood insulin levels in type 2 diabetes mellitus were triggered via the action of hypoglycemic components separated	[63]
*Capsicum frutescens* (Mirch) Solanaceae	Entire plant or Fruit	Capsaicin, protein	There is the devaluation of insulin binding on insulin receptors along with boosting insulin voiding	[51]
*Coriandrum sativum* (Coriander fruits) Umbelliferae	Seed		Blood glucose level declined including the activity of beta cells escalated thereafter augmenting insulin release	[62]
*Cuminum cyminum* (Jira) Umbelliferaceae	Seed	Geraniol, coriandrol, pinene, coriendrlyacetate	Depletion in glycosylated hemoglobin, blood urea nitrogen, and blood glucose, and at the same time serum insulin content is enhanced	[62]
*Eucalyptus globulas* (Nilgiri, Dinkum oil)	Leaf	Hydrocumin, phellandrene, and cuminaldehyde	Elevation of peripheral glucose uptake	[61,92,95]
*Curcuma longa Le*. (Turmeric) Zingiberaceae	Tuber	Citronella, camphene, pinene, cineole	Mentioned medicine showed promising outcomes in the management of diabetes	[13,62]
*Eugenia jambolana* (jamun) Myrtaceae	Dried seed and pulp	Essential oil, dimethoxy curcumin, curcumin, and Btermennone	Intensifying the emission of insulin in addition to hindrance of the liver and kidney enzyme insulinase	[96]
*Trigonella foenum* *Graecum* (Methi) Leguminosae	Seed	Oleanolic acid, ellagic acid, alpha glucosidase, Malvidin 3-laminaribiosidea and ferulic acid	Valuable discharge of insulin alongside the inducement of insulin coalescence	[51,97]
*Tinospora crispa* Menispermaceae	Stem	Nicotinic acid, coumarin, saponin-peptide esters, trigonelline, and flavonoids	Stimulation of insulin secretion on account of its anti-diabetic effect and further, there is the regulation of calcium concentration of beta cells	[51,62]
*Ocimum sanctum* L. (Tulsi) Lamiaceae	Leaf	Fraxinus coumarin alkaloids, asco acid, eugenol, and glucoside	Serum glucose level is diminished	[62]
*Lawsonia inermis* (Henna) Lythraceae	Seed and flower	Xanthones and tannin, alkaloids and fatty oil	Concentration of cholesterol, glucose, and triglycerides is depressed	[98]
*Momordica charantia* (Karela) Cucurbitaceae	Leaf	Charantin, momordic I, momordic II, and cucurbitacin B	There may be rejuvenation of moderately damaged cells along with improvement of beta cell production in the pancreas. In addition, said product contains lectin, which mimics the action of insulin	[92]
*Mangifera indica* Anacardiaceae	Leaf	Mangiferin	Intestinal absorption of glucose is decreased	[42]
*Musa sapientum* (Banana) Musaceae	Flower	Glycoside, flavonoids, and steroid	Works by simulating insulin-like action	[99]
*Tinospora Cardifolia* (Guduchi) Menispermaceae	Root, stem, and leaves	Diterpenoid lactones, alkaloid, glycosides, and steroids	Appreciable depression of blood sugar	[100]
*Psibium guajava* Myrtaceae	Fruit	Strictinin, vitamin C, quercetin, and glycon	The blood sugar level is lowered via the glycon existing in fruit	[60]
*Murraya koenigii* (Curry Leaves) Rubaceae	Leaf	Carbazole alkaloids	Declined gluconeogenesis and glycogenolysis	[101]
*Cajanus cajan* (Arhar) Fabaceae	Seed	Cajanin, cajanones, 2-2 methyl cajanone, and isoflavones	Appreciable decrease in levels of blood glucose	[76]
*Coccinia indica* (baby watermelon) Cucurbitaceae	Whole plant	Asparagine and glutamic acid	Due to repressed glucose synthesis, there is a reduction in blood glucose level	[62]
*Panex ginseng* (Ginseng) Araliaceae	Extract of root	Ginsenosides and protopanaxadiol	Decline the assimilation of glucose along with blocking the action of enzyme alpha-glycosidase	[92]
*Annona squamosa* (Sharifa) Annonaceae	Leaf extract	Moupinamide and liriodenin	Glucose tolerance is enhanced	[61]
*Punica grantum* (Pomegranate) Puniaceae	Seed extract	Punicalin and punicalagin	Blood sugar reduction	[92]

## Data Availability

The data will be available from the corresponding author up on request.
